# Cis-regulatory elements are harbored in Intron5 of the *RUNX1* gene

**DOI:** 10.1186/1471-2164-15-225

**Published:** 2014-03-24

**Authors:** Boris Rebolledo-Jaramillo, Ricardo A Alarcon, Valentina I Fernandez, Soraya E Gutierrez

**Affiliations:** 1 Departamento de Bioquimica y Biologia Molecular, Facultad de Ciencias Biologicas, Universidad de Concepcion, Concepcion, Chile

**Keywords:** *RUNX1* gene, AML, (8; 21) translocation, Non coding sequences, Enhancer modules, Chromatin structure

## Abstract

**Background:**

Human *RUNX1* gene is one of the most frequent target for chromosomal translocations associated with acute myeloid leukemia (AML) and acute lymphoid leukemia (ALL). The highest prevalence in AML is noted with (8; 21) translocation; which represents 12 to 15% of all AML cases. Interestingly, all the breakpoints mapped to date in t(8;21) are clustered in intron 5 of the *RUNX1* gene and intron 1 of the *ETO* gene. No homologous sequences have been found at the recombination regions; but DNase I hypersensitive sites (DHS) have been mapped to the areas of the genes involved in t(8;21). Presence of DHS sites is commonly associated with regulatory elements such as promoters, enhancers and silencers, among others.

**Results:**

In this study we used a combination of comparative genomics, cloning and transfection assays to evaluate potential regulatory elements located in intron 5 of the *RUNX1* gene. Our genomic analysis identified nine conserved non-coding sequences that are evolutionarily conserved among rat, mouse and human. We cloned two of these regions in pGL-3 Promoter plasmid in order to analyze their transcriptional regulatory activity. Our results demonstrate that the identified regions can indeed regulate transcription of a reporter gene in a distance and position independent manner; moreover, their transcriptional effect is cell type specific.

**Conclusions:**

We have identified nine conserved non coding sequence that are harbored in intron 5 of the *RUNX1* gene. We have also demonstrated that two of these regions can regulate transcriptional activity *in vitro*. Taken together our results suggest that intron 5 of the *RUNX1* gene contains multiple potential cis-regulatory elements.

## Background

The transcription factor RUNX1/AML1 is an important regulator of hematopoiesis and *RUNX1* gene is one of the most frequent target of chromosomal translocations in cells of the myeloid lineage [1]. Interestingly, the *RUNX1* gene covers 260 kbp of chromosome 21 but surprisingly, all genomic breakpoints for the leukemia causing translocations (8; 21) and (16;21) are found in intron 5 of the gene [2]. Presently, factors involved in maintaining the structural integrity and/or enhancing susceptibility of these regions to undergo recombination are unknown. Moreover, the breakpoint junctions are devoid of common DNA motifs that can explain the high recombination frequency observed. Interestingly however, topoisomerase II and DNase I hypersensitive sites have been found to correlate with breakpoints suggesting that chromatin organization may be responsible for, or contribute to, chromosomal translocation formation [3,4]. DNA regions that exhibit DNase I hypersensitivity have been extensively associated with the presence of cis-acting regulatory elements, including promoters, enhancers, silencers, insulators and locus control regions [5]. In fact, mapping DNase I hypersensitive sites (DHS) within nuclear chromatin is a traditional and powerful method used to identify genetic regulatory elements [5,6]. Therefore, presence of DHS in intron 5 of the *RUNX1* gene suggests that transcriptional regulatory modules maybe harbored in this gene region. In fact, well conserved and functional enhancer modules have been identified in intronic regions of the mouse *Runx1* gene [7-9]. These enhancers regulate *Runx1* expression in keratinocytes [9] and in hemogenic ECs and HSCs during early embryonic development and also in long term repopulating HSCs (LT-HSCs) in adults [7,8].

It is widely accepted that evolutionary forces drive the architecture of our genomes, and one of the cornerstones of this philosophy is that sequences that remain highly conserved between divergent organisms are likely to be functional. Genomic comparison of diverse set of vertebrate species revealed many genomic intervals that have remained conserved throughout the vertebrate lineage [10]. Some of these sequences correspond to coding genes and non-coding RNAs, however two third of them are unlikely to produce a functional transcript. Collectively these sequences are called conserved non-coding sequences (CNSs) [11]. Most of the identified conserved elements harbor transcriptional regulatory modules [12]. Therefore, comparative genomics based strategies are now being employed to predict genomic regions involved in transcriptional regulation, even in the absence of knowledge about the specific characteristics of individual cis-regulatory element [13].

In the present study, through combined application of comparative genome sequence analyses we have identified nine conserved non coding sequences present in the intron 5 of the *RUNX1* gene. Evaluation of transcriptional activity through transfection experiments of two of these sequences has shown that they can regulate transcriptional activity in a position and distance independent manner. Moreover, when their transcriptional effect was analyzed in different cell lines, these regions exhibit cell specific transcriptional regulation of a reporter gene. Taken together, our results suggest that intron 5 of the *RUNX1* gene harbor potential cis-regulatory elements.

## Results and discussion

### Identification of Conserved Non Coding Sequences (CNS) in intron 5 of the *RUNX1* gene

**Figure 1:**
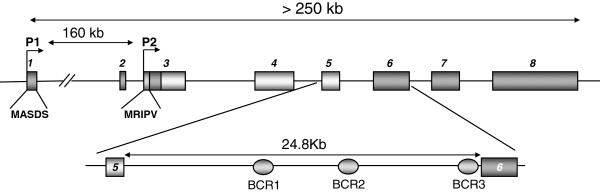
**Genomic Organization of the *RUNX1 *Gene.** Diagram of the exon-intron distribution of the *RUNX1* gene is shown. *RUNX1* proteins can be derived from two alternative promoters (P1 and P2) as indicated. Also shown is a magnification of intron 5 were the approximate location of the breakpoint cluster regions for (8;21) translocation are indicated.

All the breakpoints mapped for the (8;21) and (3;21) translocations are restricted to intron 5 of the *RUNX1* gene (Figure 1). Previous reports have shown the presence of DNaseI hypersensitive sites in this gene region [2]. It is well established that hypersensitivity to DNase I is a hallmark of DNA regions harboring cis-acting sequences such as promoters, enhancers and insulators, among others biologically active elements [5,6]. Therefore, we hypothesize that transcriptional regulatory elements maybe located in intron 5 of the *RUNX1* gene. A useful indicator to identify a sequence with functional relevance is conservation through evolution. Indeed, apart from exonic sequence, which comprise approximately 3.7% of the human genome, there are an additional 1-2% single copy conserved nongenic sequences recognizable by human-mouse comparisons [11]. In recent years, several Bioinformatics tools have been developed to aid in genome comparative analysis. In this study, in order to identify sequences in *RUNX1-*intron 5 that may have a functional role, we performed an *in silico* analysis using M-LAGAN software available at m-VISTA (http://genome.lbl.gov/vista/index.shtml) [14] looking for conserved non-coding sequences (CNS). These analyses are based on the premise that functionally significant parts of the genomic sequences evolve more slowly than their non-functional neighborhood. Initially, we carried out a multi-species sequence alignment. To this end, sequences of *RUNX1*-intron 5 from 28 different species, ranging from human to Pufferfish *Fugu rubripes*, were compared. Consistent with the evolutionary tree, we observed greater than 97% sequence conservation between human and chimpanzee (data not shown) throughout the whole gene sequence. Interestingly, comparative sequence analysis across species revealed presence of eleven highly conserved regions in *RUNX1-*intron 5. These regions show greater than 70% sequence identity and are spread throughout the intron. Moreover, they are highly conserved across mammalian species. These evolutionarily conserved genomic fragments range in size from 126 to greater than 500 base pairs.

**Figure 2:**
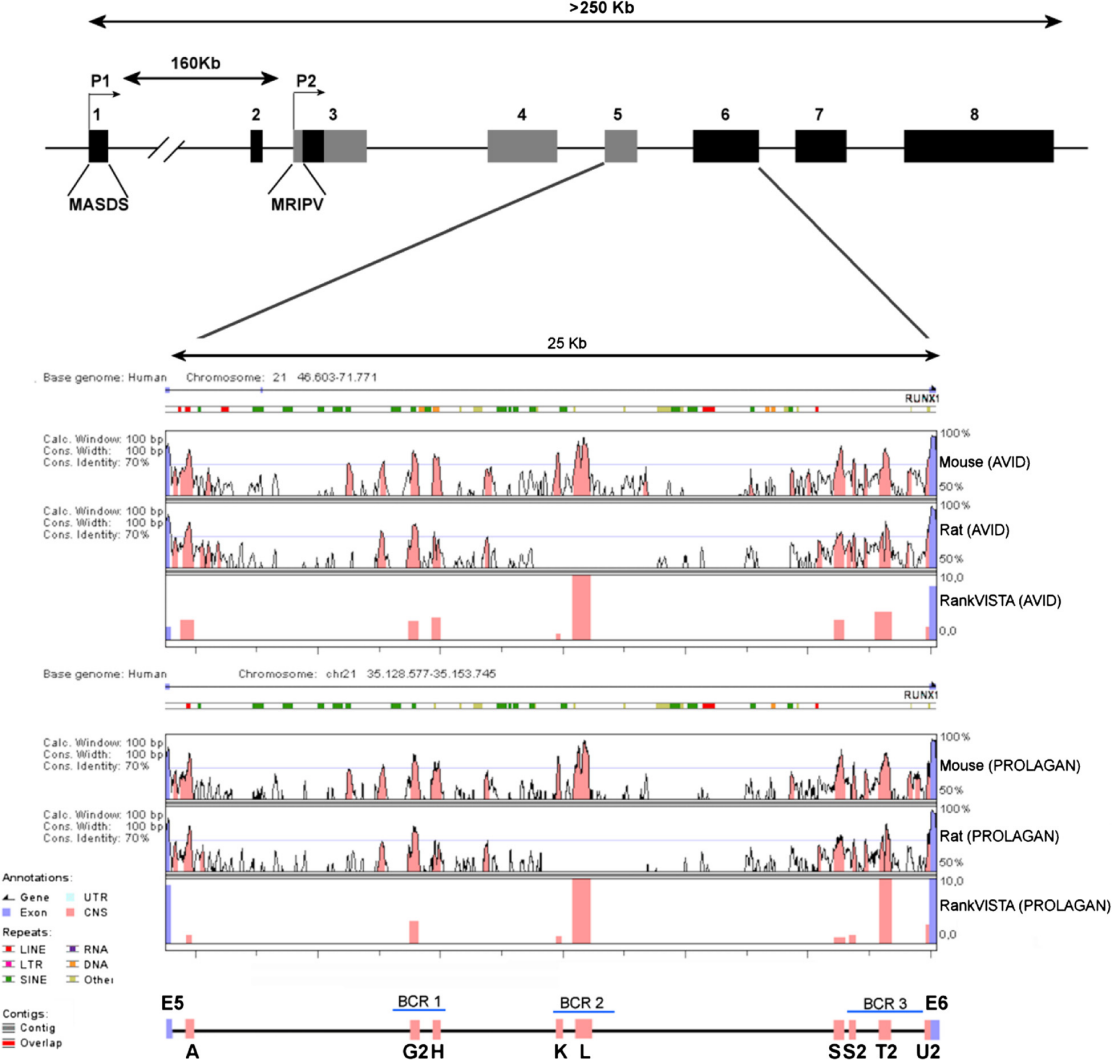
**Identification of Conserved Non-coding Sequences (CNS) in intron 5 of the *RUNX1 *Gene.** Top panel shows the genomic organization of the *RUNX1* gene. Middle panel correspond to visualization of sequence alignment outputs for comparison of intron 5 sequence among mouse-human and rat-human using AVID and PROLAGAN alignment methods respectively. Bottom panel correspond to an schematic representation of the identified CNS and also their relative position to the breakpoint cluster regions (BCRs) mapped for t(8;21) are indicated.

**Figure 3:**
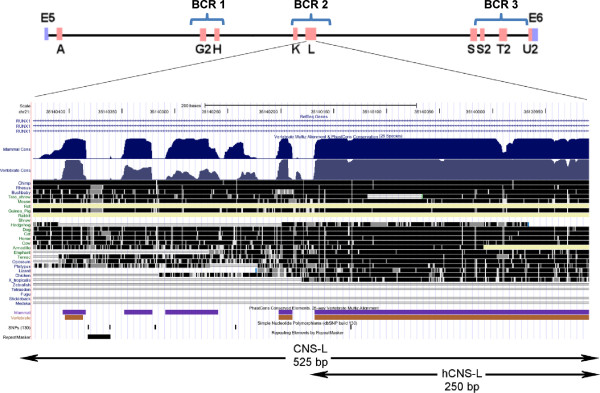
**Identification of a highly Conserved Region in CNS L.** Visualization of sequence alignment outputs for comparison of CNS L sequence among 28 vertebrate species. Dark blue indicates conservation among mammals and light blue for vertebrates in general.

We next examined in detail the conservation among mouse, rat and human in the *RUNX1*-intron 5 sequence using both AVID and PROLANGAN alignment programs for this analysis. Our results confirmed the presence of eleven conserved non-coding regions (CNS) among the alignment mouse-human and rat-human, which were predicted by the two alignment methods. Additionally, nine of the eleven regions also exhibit evolutionary significance (Figure 2); therefore we continue our analysis with these nine CNS (Table 1). Closer analyses of the CNS L show a highly conserved region of approximately 230 bp that is present not only in mammals but in all vertebrate species analyzed (Figure 3).

**Table 1:** **Evolutionary conserved non coding sequences identified in *RUNX1*-intron 5**

**CNS**	**Start**	**End**	**Length (bp)**	**% Identity**
A	35252890	35153173	284	72
G2	35145513	35145742	230	77
H	35144751	35145044	294	73
K	35140896	35141062	167	77
L	35139910	35140434	525	83
S	35131601	35131893	293	74
S2	35131200	35131298	99	73
T2	35130082	35130416	335	77
U2	35128769	35128922	154	72

A common characteristic shared by all potentially functional CNSs is that they are present in a single copy in the genome [15]. Therefore, we analyzed the representation of the identified CNSs in the genome using BLASTN program with 0.01 as E-value. The results, shown in Table 2, confirm that there are no other copies of the identified CNSs in the human genome. Indeed, these results demonstrate that there is no chance at all of randomly find CNS L and T2 sequences in the human genome (E-value = 0,00E + 00).

**Table 2:** BLASTN analysis results for CNSs

**CNS**	**Coincidence**	**Description**	**E-value**
A	refǀNT_011512.10ǀHs21_11669	Homo sapiens chromosome 21 genomic contig, reference assembly	2,00E-158
G2	refǀNT_011512.10ǀHs21_11669	Homo sapiens chromosome 21 genomic contig, reference assembly	2,00E-126
H	refǀNT_011512.10ǀHs21_11669	Homo sapiens chromosome 21 genomic contig, reference assembly	2,00E-164
K	refǀNT_011512.10ǀHs21_11669	Homo sapiens chromosome 21 genomic contig, reference assembly	7,00E-89
L	refǀNT_011512.10ǀHs21_11674	Homo sapiens chromosome 21 genomic contig, reference assembly	0,00E + 00
S	refǀNT_011512.10ǀHs21_11669	Homo sapiens chromosome 21 genomic contig, reference assembly	8,00E-164
S2	refǀNT_011512.10ǀHs21_11676	Homo sapiens chromosome 21 genomic contig, reference assembly	1,00E-43
T2	refǀNT_011512.10ǀHs21_11669	Homo sapiens chromosome 21 genomic contig, reference assembly	0,00E + 00
U2	refǀNT_011512.10ǀHs21_11669	Homo sapiens chromosome 21 genomic contig, reference assembly	3,00E-81

Interestingly, six of the identified CNSs (CNS G2, H, K, L, S2 and T2) are located in previously describe breakpoint cluster regions (BCR) involved in t(8;21) formation and two more (CNS S and U2) localize in close proximity to BCR3 (Figure 2, bottom panel). Moreover, previous work from our lab [16], have shown that in hematopoietic cells the BCRs are devoid of histone H1 and enriched in acetylated histone H3 and H4. The same regions also exhibit hypersensitivity to DNase I and Topoisomerase II [17]. All these characteristics are hallmark of transcriptionally active domains. Taken together these results suggest that intron 5 of the *RUNX1* gene harbor potential transcriptional regulatory elements.

### CNS K and CNS L regulate transcriptional activity

Transcriptional activation in higher eukaryotes frequently involves cooperative action of multiple regulatory DNA elements located at distant places [18,19]. The human genome contains several different kinds of regulatory transcriptional elements, such as promoters, enhancers, silencers and insulators among others. Analysis of the Vista enhancer database revealed that five of the nine CNSs identified in *RUNX1*-intron 5 (CNS A, G2, L, S and T2) are predicted as potential enhancers.

**Figure 4:**
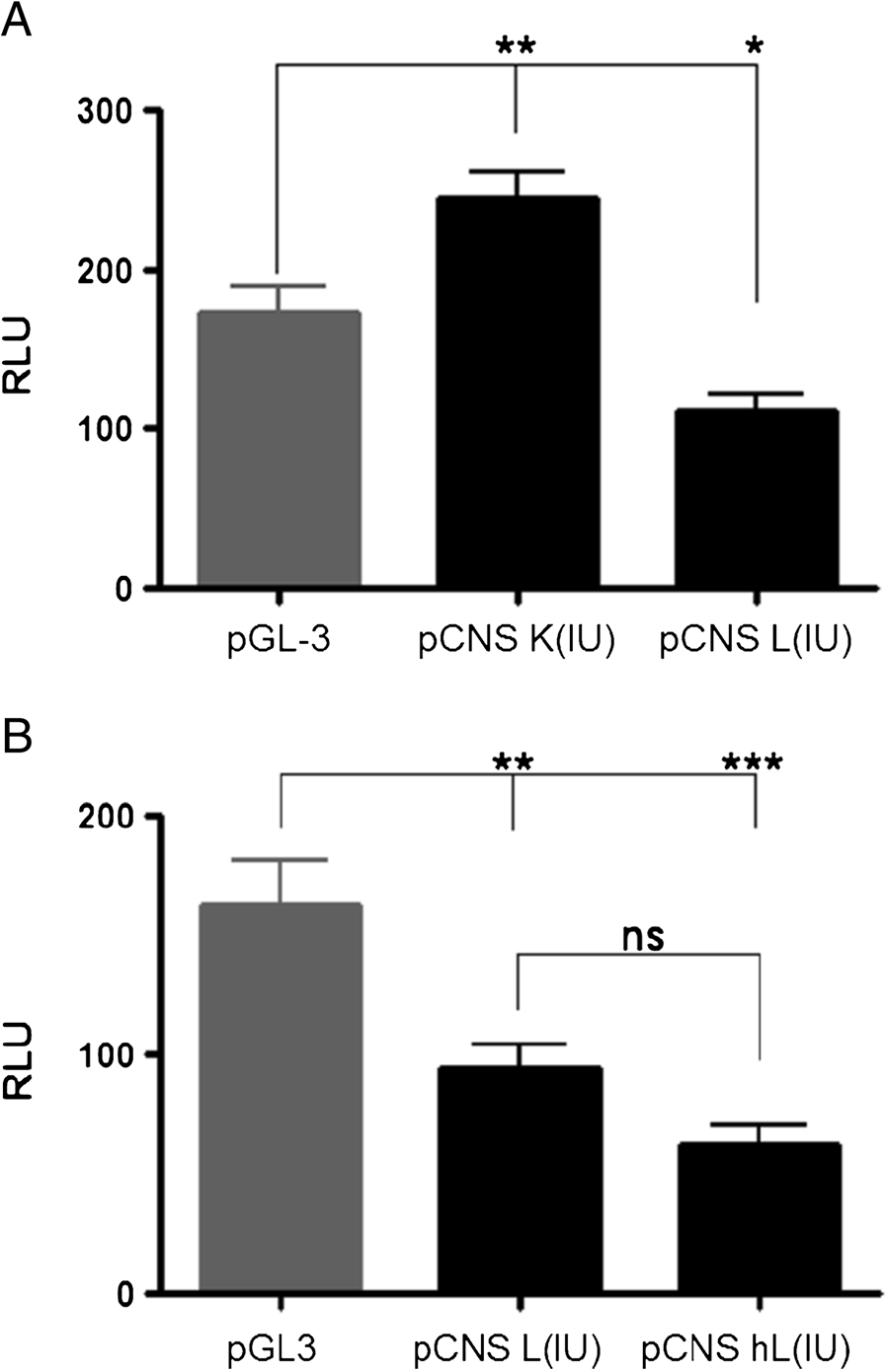
**CNS K and CNS L modulate transcriptional activity.** Transcriptional activity of CNS K, CNS L **A)** and the ultra-conserved CNS L region **B)** was evaluated. HeLa cells, cultured in 12 well dishes were transfected with the indicated constructs. Cells were harvested 24 h after transfection and luciferase and renilla activities were determined. Results are expressed as relative units of luciferase (RLU). Pooled data of at least three independent experiments are shown.

Once potential cis-regulatory elements are identified, they have to be verified experimentally; this is usually done by placing the sequences into a reporter construct that is then used for transfection in tissue culture cells or to test for expression in embryos, either as transient assays or stable transgenes. Therefore, in order to test their putative transcriptional regulatory role, we cloned two of the identified CNS (CNS K and CNS L) in pGL3-Promoter plasmid. We choose these regions because both are located in one of the breakpoint cluster regions identified for t(8;21) formation (BCR2, see Figure 2, bottom panel); they exhibit the highest conservation among rat, mouse and human (77 and 83 percent identity respectively) and CNS L also include an highly conserved region (Figure 3). The pGL3 Promoter vector contains a SV40 promoter upstream of the luciferase gene and allow for insertion of putative enhancer elements either upstream or downstream of the promoter-luciferase transcriptional unit. Initially, we cloned either CNS K or CNS L immediately upstream of the SV-40 promoter region. The resulting constructs, named CNS K(IU) and CNS L(IU) respectively, were transfected in HeLa cells and luciferase activity was determined. Our results show that both regions modulate transcriptional activity in a statistically significant manner compared to the parental pGL3 Promoter plasmid (Figure 4A). However surprisingly, they exhibit opposite transcriptional effects. In fact, while CNS L represses transcription, CNS K activates transcription of the reporter gene.

Our comparative sequence analysis identified a highly conserved region of 230 bp at the 5’end of CNS L, which is conserved between mammals and vertebrates (Figure 3). In order to evaluate if this region exhibit differential regulatory activity, we also cloned it in pGL 3 Promoter vector (pCNS hL(IU)) and compared its transcriptional effect to the full length CNS L. Our results show that both regions repress transcription of the reporter gene and that there are no statistically significant differences in their effect (Figure 4B)

Taken together our results demonstrate that CNS K and CNS L can perform a transcriptional regulatory function.

### CNS K and CNS L are putative enhancer modules

**Figure 5:**
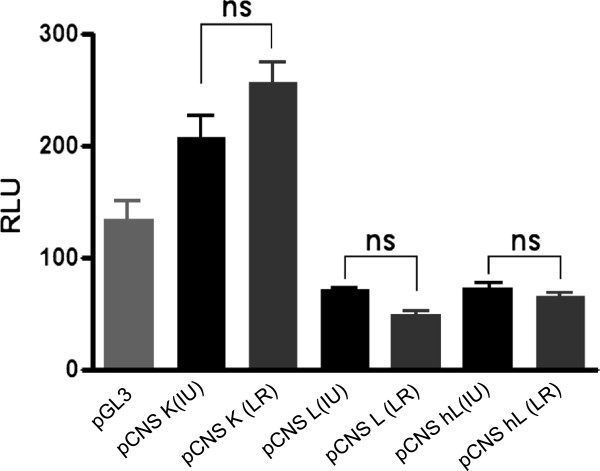
**CNS K and CNS L effect is position and orientation independent.** HeLa cells, cultured in 12 well dishes were transfected with the indicated constructs. Cells were harvested 24 h after transfection and luciferase and renilla activities were determined. Results are expressed as relative units of luciferase (RLU). Pooled data of at least three independent experiments are shown.

A hallmark of the enhancers is that they act as regulatory modules independent of orientation and distance to the promoter which activity they modulate [5]. Therefore, to test if CNS K and CNS L present this characteristic, we generated constructs where each of the CNSs was cloned downstream of the reporter coding sequence. In these constructs, named CNS K(LR) and CNS L(LR), the CNS not only is located far away from the promoter, but it can also be considered that is in reverse orientation with respect to the SV40 promoter. Our results show that both CNSs exhibit exactly the same transcriptional effect, independent of the distance, or the orientation, in which the CNSs are present with respect to the promoter (Figure 5, compare pCNS K(IU) with pCNS K(LR), pCNS L(IU) with pCNS L(LR) and pCNS hL(IU) with pCNS hL(LR), respectively).

**Figure 6:**
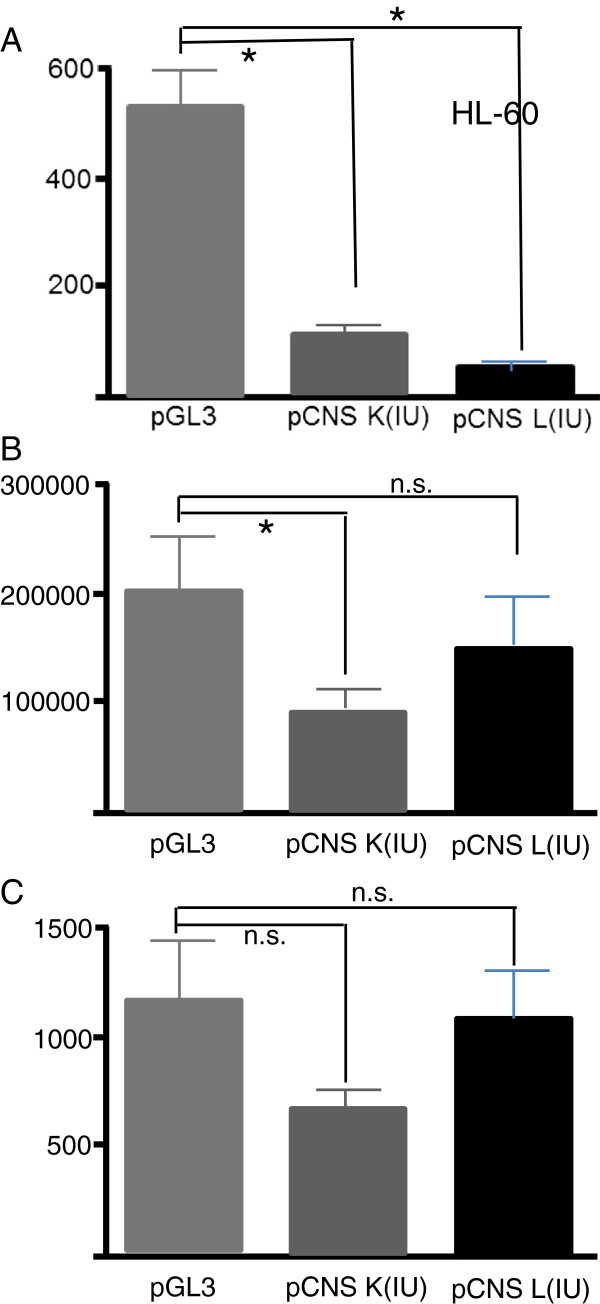
**CNS K and CNS L exhibit cell-type specific activity.** Cells, cultured in 12 well dishes were transfected with the indicated constructs. Cells were harvested 24 h after transfection and luciferase and renilla activities were determined. Results are expressed as relative units of luciferase (RLU) for HL-60 **(A)**, Jurkat **(B)** and HepG2 **(C)** cells. Pooled data of at least three independent experiments are shown.

Another characteristic of the enhancer modules is that they exhibit tissue or cell specific activity. Therefore, to test if this was also a property exhibited by CNS L and CNS K, we transfected the CNS K(IU) and CNS L(IU) in three different cell lines: HL-60, Jurkat and HepG2. According to our previous results, when transfected in HeLa cells CNS K and CNS L exhibit differential transcriptional regulation activity with CNS K activating and CNS L repressing transcription (Figure 4A). Surprisingly, in the hematopoietic cells HL-60 and Jurkat, both CNSs repressed transcription of the reporter gene (Figure 6A and B, respectively). However, in HepG2 cells neither CNS K nor CNS L exhibit a statistically significant effect in the transcriptional activity of the reporter gene (Figure 6C).

An additional level of control exerted by enhancers, and other cis-regulatory sequences, is the three dimensional organization of the genome in the nucleus. In fact, the genome of higher eukaryotes appears precisely organized at the individual chromosome level as well as the total number of chromosomes. For instance, each chromosome occupies a specific region in the nucleus named the chromosomal territory [20]. Moreover, the precise location of a given genomic region, or a chromosome territory, depends on the cell-type analyzed [20,21]. Therefore, a direct functional connection between gene nuclear localization and activity has been suggested. For instance, the differentiation of hematopoietic progenitors into either erythroid cells or neutrophils is associated with differential spatial relocation of chromosome domains in the two cell types, which relates to differentially expressed genes [22]. This nonrandom nuclear position may also be relevant in chromosomal translocation formation, because for two DNA fragments to be joined they must necessarily come in close proximity of each other. For example, the BCR and ABL genes, encoded in chromosomes 9 and 22 respectively, whose translocation leads to a fusion protein involved in leukemia, are located in close proximity in normal hematopoietic cells at much higher frequency than would be expected based on a random distribution [23,24]. Therefore, presence of regulatory modules in the breakpoint clusters regions involved in chromosomal translocations may be relevant at least for recombinant partner selection.

Our results demonstrate that, in an hetelogous system, both CNS K and CNS L regulate transcriptional activity independent of distance and orientation respect to the promoter and that their function is cell specific. Taken together these results suggest that CNS K and CNS L may act as cis-regulatory elements *in vivo*.

## Conclusions

In this report we have analyzed the intron 5 of the *RUNX1* gene where we have identified, through sequence comparison among different species, nine conserved non coding sequences. Using transient transfection assays we have also demonstrated for two of these CNSs (CNS K and CNS L) that they regulate transcription in a distance and orientation independent manner and that this effect is cell type-dependent. In eukaryotes, transcriptional regulation tends to involve combinatorial interactions between several transcription factors, which allow for a sophisticated response to multiple conditions in the cellular environment [25,26]. Furthermore, shared regulatory sequences impose genome architecture. In fact, two or more genes regulated together by the same sequence cannot be separated, for instance by translocation or inversion, without severely affecting their spatiotemporal expression pattern. Interestingly, eight of the nine conserved region that we have identified are either in or very close to a breakpoint cluster region involved in t(8; 21) formation. This association strongly suggests a role for CNSs in chromosomal translocation either by facilitating DNA double strand break formation, for instance by establishing an open chromatin conformation in these regions, or by determining a specific subnuclear localization and therefore influencing the selection of the recombination partner.

## Methods

### Cell cultures

The hematopoietic cell lines HL-60 and Jurkat were cultured in RPMI media supplemented with 10% fetal bovine serum. HeLa and HepG2 cells were grown in DMEM supplemented with 10% FBS. All cells were cultured at 37°C and with 5% CO_2_.

### Identification of CNSs

Ensembl database was used to obtain the latest version and collect the domains composition for each of the genomes analyzed. Human genome sequence was used as the reference genome in VISTA analysis. *RUNX1* gene homologous genomic regions were used as input to the MLAGAN multiple sequence alignment toolkit in order to generate an alignment with mouse and rat genomes using default parameters as previously described [14].

### Evaluation of CNS transcriptional activity

The *RUNX1*-intron 5 CNS test fragments were designed cloning the corresponding entire conserved non coding sequences as taken manually from the Vista browser [27]. The fragments were amplified from HL-60 human promyeloid cells genomic DNA and cloned in pGL3 Promoter vector (Promega, USA). This construct contains luciferase reporter gene and SV-40 minimal promoter. The reporter plasmid for each element (200 ng) was transiently transfected in the different cell lines analyzed. Briefly, the cells in each well (12-well plate, 200,000 cells/well) were transfected with SatisFaction (Stratagene, USA.) according to manufacturer’s protocol. Renilla luciferase (5 ng) was used to correct for transfection efficiency. 24 h post transfection, Luciferase activity was measured using Dual-Luciferase Reporter Assay System (Promega, USA) according to manufacturer’s protocol. Data from at least three independent experiments was collected, corrected by Renilla activity and expressed as relative light units (RLU).

## Competing interests

The authors declare that they have no competing interests.

## Authors’ contributions

BR participates in the conception and design of the study, carried out the sequence alignment, part of the molecular studies and the analysis and interpretation of data. RA and VF carried out part of the molecular studies, performed the statistical analysis and contribute to the analysis and interpretation of data. SEG conceived of the study, and participated in its design and coordination and wrote the manuscript. All authors read and approved the final manuscript.

## Authors’ information

BR is currently enrolled at the PhD in Bioinformatics and Genomics Program at the PennState University, USA. RA is a graduate student of the Cell and Molecular Biology PhD Program at Facultad de Ciencias Biologicas, University of Concepcion, Chile.
